# Investigating the association between resilience and impostor syndrome in undergraduate nursing and medical students: a cross-sectional study

**DOI:** 10.25122/jml-2024-0160

**Published:** 2024-09

**Authors:** Amal Ibrahim Khalil, Ruba Alharbi, Hadab Al Qtame, Raneem Al Bena, Muhammad Anwar Khan

**Affiliations:** 1 Psychiatric and Mental Health Nursing Department, College of Nursing, King Saud Bin Abdul-Aziz University for Health Sciences, Jeddah, Saudi Arabia; 2 King Abdullah International Medical Research Center, Jeddah, Saudi Arabia; 3 Faculty of Nursing, Menoufiya University, Shebin Elkom, Egypt; 4 College of Nursing, King Saud Bin Abdul-Aziz University for Health Sciences, Jeddah, Saudi Arabia; 5 Medical Education Department, College of Medicine, King Saud Bin Abdul-Aziz University for Health Sciences, Jeddah, Saudi Arabia

**Keywords:** impostor syndrome, resilience, undergraduate nursing and medical students, well-being, cross-sectional study, mental health, academic performance, self-efficacy

## Abstract

Impostor syndrome is prevalent among undergraduate nursing and medical students. Resilience is the ability to adapt and rebound from challenges, which is crucial for student well-being and academic success. Exploring the connection between impostor syndrome and resilience is essential to support students effectively. This cross-sectional study investigated the relationship between resilience and impostor syndrome among undergraduate nursing and medical students at King Saud Bin Abdulaziz University for Health Sciences. A total of 300 students were recruited using a convenience sample and completed self-reported questionnaires assessing resilience and impostor syndrome between September 2022 and March 2023. Various bias mitigation strategies were employed to ensure data accuracy and reliability, such as anonymous data collection and validated scales. The results indicated that less than half of the participants experienced impostor syndrome. Among nursing students, 41.7% were classified as severe, 37.7% as moderate, and 13.0% as intense impostors. For medical students, 4.6% were mild, 4.6% moderate, 4.5% severe, and 4.5% intense impostors. Mean resilience scores were 24.3 ± 7.15 for nursing students and 25.6 ± 7.22 for medical students. A significant negative correlation was found between resilience and impostor syndrome scores (r = -0.220, *P* < .001). Regression analysis indicated that resilience significantly predicted impostor syndrome, with higher resilience associated with lower levels of impostor syndrome. These findings highlight the importance of resilience in mitigating impostor syndrome among nursing and medical students. Building resilience through interventions may be beneficial in promoting student well-being and academic success. Future research should explore the effectiveness of such interventions and identify other factors contributing to impostor syndrome among healthcare students.

## INTRODUCTION

Imposter syndrome refers to a collection of emotions characterized by feelings of inadequacy and is accompanied by psychological symptoms such as persistent anxiety, low self-esteem, internal conflict between feelings of inferiority and superiority, negative self-perception, and fear of being exposed [1]. Resilience is defined as the ability to successfully adapt and achieve positive outcomes in the face of challenging or threatening circumstances [2]. It encompasses more than just overcoming anxiety; it also encompasses a heightened capacity to recover from traumatic experiences [3].

Throughout their college years, undergraduate nursing and medical students enthusiastically pursue their goals, striving to become competent health workers. The university consistently and sincerely supports these endeavors. As they progress, these students grow in confidence and develop the ability to cope with crises. Celebrating their achievements with pride and supported by their surroundings, they make the most of every opportunity [4].

However, the healthcare environment is filled with stressors, and these students often find it challenging to maintain inner peace. These are people like any other college students – who want and seek to do their best and succeed. However, managing and developing resilience to stressful situations is difficult, especially during high stress [5]. Coping with stress and building resilience are complex processes that involve various brain regions and systems. These systems interact differently and are influenced by genetic, epigenetic, and environmental factors, some of which may suppress or enhance others [6].

Impostor syndrome is common, and one may not feel as confident as it appears on the outside. They may struggle to acknowledge their accomplishments and attribute them to luck rather than their abilities. Constant competition among peers can contribute to these negative thoughts, making it difficult for them to shake off their self-doubt [7]. Impostor syndrome (IS) significantly impacts various professional dimensions, including the care and treatment of patients, communication with colleagues, teamwork, and overall professional performance. Individuals with IS may struggle to acknowledge their accomplishments, attributing their success to luck rather than their abilities, which can undermine their confidence and effectiveness in professional settings [2].

Imposter syndrome, also known as the imposter phenomenon (IP), affects high-achieving individuals and leads them to perceive themselves as less intelligent than others perceive them to be despite having identifiable accomplishments [8]. Those experiencing IP create various ways to undermine evidence contradicting their belief that they are not as smart as others think they are [6]. They feel compelled to find alternative explanations for their achievements, attributing their success to factors other than their competence, even to convince themselves that they have deceived anyone who sees them as capable [9].

Individuals with imposter syndrome have a persistent feeling of not belonging, being overvalued, and fearing being exposed to fraud. As a result, they work tirelessly, intensifying their imposter phenomenon and creating a vicious cycle. Clance and Imes [8] found that repeated successes alone did not stop this pattern. Despite their achievements, they feel a sense of emptiness, and the positive feelings are short-lived because the core belief of fraud remains unchanged. Clance and Imes [9] observed that imposter syndrome is influenced by familial relationships, social pressures, and stereotypes, leading to a strong desire to please others. These individuals exert tremendous efforts to achieve success and prove to others and themselves that they are intelligent and deserving of their accomplishments [7]. However, they experience a profound disappointment when they fall short of their self-imposed, extremely high expectations and aspirations [1].

Resilience reflects a person’s ability to transform hardships and difficult experiences into educational ones [9]. Nursing students try to navigate their way through their college years while struggling with a lack of will to care because of academic difficulties and the emotional and physical exhaustion of their day-to-day lives as nursing majors [7]. Furthermore, medical students' resilience plays a role in reducing the physical toll associated with the stress of their training, yet this demanding process can still negatively impact their quality of life [10]. According to Maqsood *et al*. [11], medical students feel burdened by their accomplishments and undeserving of applause. Yildirim and Celik Tanriverdi [12] found a significant positive relationship between resilience acquired from social support and fulfillment and quality of life. In contrast, Safaryazdi [13] reported that individuals with impostor syndrome tend to have a strong inclination toward self-criticism and feelings of inadequacy. Additionally, Kleitman [1] demonstrated that extreme parental support, combined with a lack of emotional care and intimacy within the family, are the strongest predictors of impostor syndrome. Managing feelings of impostor phenomenon is important and increasingly recognized, particularly in the medical field, because it has been linked to mental health problems such as depression and the three domains of burnout [4], decreased self-esteem [14], approval-seeking, perfectionism [15], and anxiety [16]. However, contrary to these findings, Alrayyes *et al*. [6] did not find a significant association between the impostor phenomenon and these issues. Research on the impostor phenomenon has also explored its relationship with sociodemographic factors such as gender. Some studies suggest that it is significantly higher among women than men [4,17], and a few studies found the opposite [9,16]. Other studies reported no significant difference between genders [14,18].

Recent studies have demonstrated the significance of this phenomenon by showing troubling results. A study in Saudi Arabia identified that 57.8% of young adults had IP [4]. A study in the United States reported that 97% of medical students experienced moderate-to-intense feelings of imposter syndrome, in which 38.4% had moderate imposter syndrome, 49.6% had frequent IP, and 8.8% experienced intense IP [19]. Another recent study in Canada revealed that approximately 75% of undergraduate students have IP [17] and how IP may impact students’ professional aims [19]. Moreover, in a recent study, Smith *et al*. [20] underscored the significance of a supportive educational environment in fostering resilience among nursing students. Their research indicates that mentorship programs and peer support networks are vital in helping students manage stress and maintain their overall well-being. Additionally, Parkman [21] delved into the impact of impostor syndrome on medical students, revealing its detrimental effects on academic performance and heightened psychological distress. Furthermore, Villwock *et al*. [22] found a correlation between impostor syndrome, increased burnout rates, and reduced professional satisfaction among medical residents.

There has been a noticeable increase in both the prevalence of imposter syndrome and the recognition of the role resilience plays in maintaining positive mental health. As research on the topic is scarce, particularly in the Kingdom of Saudi Arabia, we conducted the first study on nursing students at King Saud bin Abdulaziz University for Health Sciences. We aimed to determine the prevalence of imposter phenomena among nursing and medical students and assess the impact of resilience on this phenomenon. Through our study, we were able to compare the total scores of resilience and imposter syndrome among nursing and medical students, examine the connection between resiliency scores and imposter phenomena, and identify a correlation between resiliency, imposter syndrome, and the sociodemographic background of nursing and medical students.

### Theoretical framework

The relationship between resilience and impostor syndrome among undergraduate nursing and medical students can be comprehensively understood by considering Bandura's Social Cognitive Theory [23] and Lazarus and Folkman's transactional model of stress and coping [24]. Bandura's Social Cognitive Theory underscores the interplay between personal factors, environmental influences, and behavioral outcomes. Resilience, characterized by an individual's ability to cope with stress and rebound from adversity [23], is influenced by personal factors such as self-esteem, self-confidence, and self-efficacy [25]. Higher levels of resilience foster positive traits such as adaptability, perseverance, and self-assurance, which may serve as protective factors against the development of impostor syndrome [25]. Conversely, impostor syndrome, typified by feelings of inadequacy and self-doubt [6], may be influenced by negative traits like perfectionism and fear of failure [25].

Furthermore, the application of Lazarus and Folkman's transactional model of stress and coping sheds light on how individuals manage stressors and adapt to challenging circumstances [24]. Resilient individuals with effective coping strategies, such as problem-solving skills, social support, and positive thinking, are better positioned to navigate stressors [25]. This effective coping may act as a buffer against the onset and exacerbation of impostor syndrome. Conversely, individuals grappling with impostor syndrome may resort to maladaptive coping mechanisms, exacerbating their feelings of inadequacy [24].

In addition to individual factors, the academic environment significantly influences students' experiences and psychological well-being. High academic demands and competitive learning environments can contribute to the emergence of impostor syndrome [8]. Conversely, a supportive academic environment that nurtures resilience through mentorship and constructive feedback can positively shape students' self-perception [19]. Mediating factors such as self-reflection, coping strategies, social support, and academic self-concept further modulate the relationship between resilience and impostor syndrome among nursing and medical students [25]. By integrating Bandura's Social Cognitive Theory and Lazarus and Folkman's transactional model of stress and coping, we comprehensively understand the complex interplay between resilience, impostor syndrome, and the academic environment among undergraduate nursing and medical students.

To improve the understanding, a fourth Integrated Model of Impostor Resilience (IMIR) was utilized. This model elaborates on existing theories but introduces novel elements that are especially relevant in the academic arena of medicine and nursing students.

**Figure 1:**
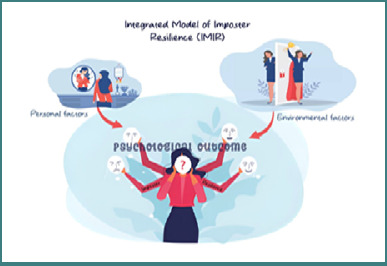
Components of the Integrated Model of Imposter Resilience (IMIR)

The diagram in Figure 1 illustrates the Integrated Model of Imposter Resilience (IMIR), offering insights into how personal and environmental factors contribute to imposter resilience.

These are the key points:

#### I. Individual factors

These encompass cognitive processes (represented by a brain icon) and emotional stability or satisfaction (depicted by a heart with a checkmark).

- Self-assertion and confidence: Drawing on Bandura's theory, the IMIR model explores how self-efficacy influences resilience concerning imposter syndrome.
- Personality traits: The model identifies three traits linked to both imposters and resilience, including perfectionism, failure avoidance, and flexibility.
- Coping strategies: By referencing Lazarus and Folkman's framework, the model distinguishes between adaptive (e.g., problem-solving, seeking support) and maladaptive coping mechanisms.

#### II. Environmental factors

These elements comprise external validation, accomplishments, and acknowledgment, represented by a trophy, podium, and confetti. The theory indicates that a fusion of internal self-perceptions and external validation impacts psychological outcomes associated with imposter syndrome within the academic setting. The IMIR model underscores the significance of academic demands, competition, and the support system, encompassing mentorship and peer support. The theory delves into the consequences of positive versus negative feedback on students' self-image and resilience, which can arise from teachers, advisors, and student mentors.

#### III. Psychological outcomes

The theory investigates the influence of personal attributes and characteristics with environmental factors on psychological outcomes. These factors include resilience, the ability to bounce back from challenges and preserve psychological well-being, and are influenced by individual and environmental elements. Furthermore, the theory addresses impostor syndrome, characterized by feelings of accomplishment and is tempered by resilience and coping strategies.

The proposed model structure will have the following interactions and pathways:

- **Direct pathways:** High self-efficacy and confidence → Increased resilience [23].
  High perfectionism and fear of failure → Increased impostor syndrome [25].
- **Moderated pathways:** Academic support and mentorship moderate the relationship between perfectionism and impostor syndrome [22]. Effective coping mechanisms moderate the impact of academic stress on both resilience and impostor syndrome [26].
- **Feedback loop:** Resilience influences coping strategies, which affect the perception of stress and the likelihood of experiencing impostor syndrome [27]. Experiences of impostor syndrome can reduce resilience over time if not mitigated by effective coping and support [28,29].

#### IV. Mediating factors

These include self-reflection to promote self-awareness and growth, influencing resilience and impostor feelings [29], and social support, which acts as a buffer against stress, enhancing resilience and reducing the impact of impostor syndrome [30,31]. By integrating these elements of the IMIR model, our study can present a novel and comprehensive model that enhances the understanding of how impostor syndrome and resilience interact among undergraduate nursing and medical students, adding significant value to the scientific community. Hence, tackling the impostor phenomenon necessitates individual factors and a broader contextual and environmental consideration. With the appreciation of these cultural determinants, we can devise more impactful interventions and bolster support for individuals grappling with impostor syndrome.

### Aim of the study

The main aim was to investigate the association between imposter syndrome and resilience among undergraduate nursing and medical students. More specifically, we aimed to:

- Assess the levels of resilience and IS among undergraduate nursing and medical students.
- Examine the correlation between resilience levels and impostor syndrome scores among undergraduate nursing and medical students.
- Investigate how the participants’ demographic backgrounds relate to impostor syndrome among undergraduate nursing and medical students.
- Identify the differences between nursing and medical students' resilience and impostor syndrome experiences.

### Significance of the study

The significance of the study lies in its exploration of the relationship between resilience and impostor syndrome among nursing and medical students, addressing a critical aspect of healthcare education. A study conducted in Saudi Arabia identified that 57.8% of young adults in the Kingdom had IP [4]. Research in the United States found that 97% of medical students encountered moderate to intense levels of impostor syndrome, 38.4% experienced moderate IP, 49.6% frequent IP, and 8.8% intense IP. Additionally, a recent study in Canada disclosed that around 75% of undergraduate students exhibit symptoms of IP. The impact of IP on students' professional aspirations remains inadequately understood. Therefore, understanding this relationship will offer insights into the psychological well-being and self-perception of future healthcare professionals while also contributing to existing literature on these constructs. Moreover, the findings may inform the development of targeted interventions and support programs to foster students' well-being and professional growth, thus enhancing the overall academic environment and student experience.

## Material and Methods

### Participants

This study included all undergraduate nursing and medical students from the third to sixth year, both men and women, at King Saud bin Abdulaziz University for Health Sciences (KSAU-HS). Participants were asked to complete a survey assessing their levels of resilience and impostor syndrome. During the fall semester of the 2022-2023 academic year, approximately 375 senior and junior nursing students and 300 medical students were eligible for the study. Purposive and convenience sampling techniques were used to collect data from students in both the Colleges of Nursing and Medicine, with the following inclusion criteria:

1. Undergraduate nursing and medical students in their third to sixth year
2. Both genders
3. Enrolled at KSAU-HS during the academic year 2022-2023
4. Agree to participate in the survey.

The exclusion criteria comprised all students from the first and second years, graduate or postgraduate students not enrolled at KSAU-HS during the academic year 2022-2023, and students who did not consent to participate in the study.

### Sample limitations

It was acknowledged that the study was conducted on a sample of undergraduate students from a single university, which limits the ability to fully represent the range of experiences with imposter syndrome or resilience in other cultural or academic environments. This focus allows for rich, context-specific insights but might not generalize to other populations. Future work should extend this to more diverse samples at multiple universities representing different cultural backgrounds for a richer understanding of these phenomena in various contexts.

### Sample size calculation

Using the Roe-soft program, it was determined that a minimum sample size of 246 out of 675 nursing and medical students was necessary to achieve a 95% confidence level with a ± 5% margin of error.

### Data collection tools

The research employed self-reported questionnaires, specifically the Conner-Davidson Resilience Scale (CD-RISC) and the Clance Impostor Phenomenon Scale (CIPS), which were validated tools endorsed by their developers. Despite their reliability and validity, self-report surveys can be influenced by biases like social desirability and inaccurate self-assessment. To counter these biases, the study guaranteed respondents' anonymity and confidentiality to promote truthful and precise self-disclosure. Moreover, clear guidance and definitions were provided to reduce confusion and improve the accuracy of self-evaluations.

The tools of the current study consisted of three main parts.

1. Sociodemographic characteristics: The questionnaire was intended to elicit sociodemographic data about the study participants, such as their age and gender.
2. Clance Impostor Phenomenon Scale (CIPS): The Clance Impostor Phenomenon Scale (CIPS) questionnaire, developed by Clance in 1985, was utilized to identify symptoms of the impostor phenomenon. Freeman *et al*. [32] demonstrated the high internal consistency of the CIPS, with a Cronbach's alpha coefficient of 0.96, indicating strong reliability. This questionnaire comprised 20 items that assessed three dimensions: (1) fear of evaluation, (2) fear of not replicating success, and (3) fear of being less competent than others. Respondents rated their agreement on a 5-point Likert scale ranging from 1 (not at all true) to 5 (very true). The total score ranged from 20 to 100, with mild (20-40), moderate (40-60), severe (60-80), and intense (>80) to determine the severity of IP symptoms. The cutoff point of 62/100 was used to identify the presence of IP [32].
3. Connor-Davidson Resilience Scale (CD-RISC-10): The CD-RISC-10, a shortened version of the original 25-item scale, was used to assess resilience. Aloba *et al*. [33] assessed the CD-RISC-10 among nursing students in Southwestern Nigeria, demonstrating satisfactory validity and good reliability with a Cronbach's alpha coefficient of .81. According to the developers of the scale [34,35], it has strong validity, reliability, and practicality compared to other versions of resilience scales. The scale primarily measures hardiness and includes items related to flexibility (Items 1 and 5), self-efficacy (Items 2, 4, and 9), emotion regulation ability (Item 10), optimism (Items 3, 6, and 8), cognitive focus and attention maintenance under stress (Item 7). Each statement is rated on a five-point scale from 0 to 4, where 0 indicates that the statement is not true at all, and 4 represents the statement being true nearly all the time. The total score was obtained by summing the scores of all 10 items, ranging from 0 to 40. Higher scores indicate greater resilience, while lower scores indicate lower resilience or greater difficulty in recovering from adversity. Interpretation of the scale scores was performed based on quartiles. The score distribution was divided into quartiles: the lowest quartile ranged from 0 to 29, the second quartile ranged from 30 to 32, the third quartile ranged from 33 to 36, and the top quartile ranged from 37 to 40. Scores in the lowest or second quartile may indicate difficulties coping with stress or returning from adversity.

### Validity and reliability in a specific context

Although the instruments used are validated by the original authors, who stated that the English version of the scales used in the current study has structural validity and reliability, it is crucial to assess their validity and reliability within the specific target population. We conducted a preliminary study to evaluate the consistency of these measures in the context of Saudi Arabia. The reliability scores obtained for the CD-RISC were 0.89 and 0.82 CIPS in our pilot investigation, which was in line with those documented in past studies.

### Data collection procedure

To facilitate the distribution of the questionnaires, correspondence was sent to each college dean. The questionnaires were converted to Google Forms in English, as all subjects in both colleges were taught in English. The links to the surveys were then shared with the deans for dissemination to students, either manually or via relevant social networks. However, the data collection encountered significant challenges due to a very poor response rate, which required more than five follow-up notifications and a physical presence to encourage students to complete the surveys. This process spanned 7 months, from September 2022 to March 2023. WhatsApp groups, college boards, emails, and websites were utilized to inform and announce the study. Out of 675 nursing and medical students approached, only 300 ultimately completed the questionnaire.

### Statistical analysis

The data was analyzed and coded using version 20.0 of SPSS. Descriptive statistics such as frequencies, percentages, means, and standard deviations were used to present the variables. Categorical data was compared using the Chi-squared or Fisher exact test. For comparison between numerical and categorical data, the student's t-test and analysis of variance (ANOVA) were applied. The Pearson correlation and multiple regression tests were used to determine the correlation value between the variables under study. The significance level was tested and adjusted at *P* <0.05.

## RESULTS

Table 1 presents the demographic data of the 300 students from the nursing and medical fields included in the study. The respondents were 21.4 ± 2.2 years old and had a grade point average (GPA) of 4.3 ± 0.5 out of 5. The majority were women (238 or 79.3%) and came from the Western region of Saudi Arabia (237 or 79%). Two-thirds of the students pursued a degree in nursing (202 or 67.3%), while the remaining students studied medicine (98 or 32.7%). 96.7% of the participants were not married, and the majority had a medium socioeconomic status (265 or 88.3%) compared to only 0.8 with a high economic status.

**Table 1:** Demographic characteristics of the participants (*n* = 300)

	N	Mean	SD
**Age**	300	21.4	2.24
**Academic GPA**	300	4.3	0.47
		*n* = 300	%
**Gender**			
	Male	62	20.7
	Female	238	79.3
**Region**			
	Western	237	79.0
	Eastern	38	12.7
	Central	15	5.0
	Southern	8	2.7
	Others	2	0.7
**Academic specification**			
	Nursing	202	67.3
	Medicine	98	32.7
**Academic year**			
	3^rd^ academic year	131	43.7
	4^th^ academic year	110	36.7
	5^th^ academic year	28	9.3
	6^th^ academic year	31	10.3
**Marital status**			
	Single	290	96.7
	Married	4	1.3
	Divorced	2	.7
	Other	4	1.3
**Socioeconomic status**			
	Low	11	3.7
	Medium	265	88.3
	High	24	8.0

**Figure 2:**
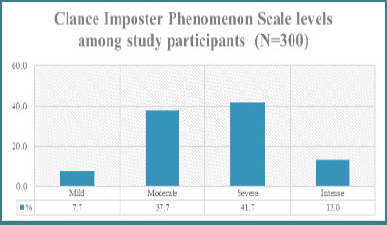
Clance Imposter Phenomenon Scale (CIPS) severity levels

As shown in Figure 2, 41.7% of participants had symptoms categorized as severe impostor syndrome, while 37.7% and 13.0% had moderate and intense symptoms, respectively. A minority (7.7%) had mild symptoms.

**Figure 3:**
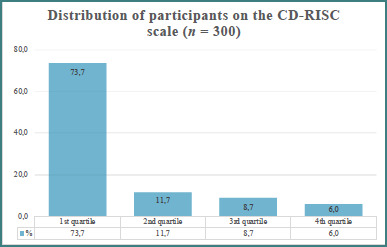
Connor-Davidson Resilience Scale (CD-RISC) severity levels

Based on Figure 3, 73.7% of participants scored in the lowest quartile of resilience, indicating the 1^st^ quartile. The remaining participants were distributed as follows: 11.7% in the 2^nd^ quartile, 8.7% in the 3^rd^ quartile, and 6.0% in the 4^th^ quartile.

The study assessed the severity of impostor syndrome using the CIPS and resilience using the CD-RISC. The overall mean score for the CIPS, calculated from the 20 items in the questionnaire, was 3.1 ± 0.7 out of 4. On the other hand, the overall mean score for the CD-RISC, consisting of 10 items, was 2.5 ± 0.7 out of 3.

Table 2 indicates that a significant number of students (238 or 79.4%) reported moderate to severe levels of impostor phenomenon, which is a concerning finding. Additionally, 256 students (85.4%) scored in the first and second quartiles of the CD-RISC scale, indicating a lower level of resilience. It is important that there was no significant difference in the severity levels of impostor syndrome and resilience across the participants. In summary, the study revealed a considerable prevalence of the impostor phenomenon and a relatively low level of resilience among students, with no significant difference observed in the severity levels of the two scales.

**Table 2:** Distribution of resilience and imposter mean values across different severity levels (*n* = 300)

	*n*	Mean	SD	95% CI		*P*
**Clance Imposter Phenomenon Scale**						
**Mild**	23	4.2	0.63	(3.88	4.42)	0.135
**Moderate**	113	4.4	0.42	(4.30	4.46)	
**Severe**	125	4.3	0.46	(4.20	4.37)	
**Intense**	39	4.3	0.51	(4.13	4.47)	
**Connor-Davidson Resilience Scale**						
**1^st^ Quartile**	221	4.3	0.47	(4.25	4.37)	0.702
**2^nd^ Quartile**	35	4.3	0.49	(4.12	4.46)	
**3^rd^ Quartile**	26	4.3	0.42	(4.11	4.45)	
**4^th^ Quartile**	18	4.4	0.52	(4.17	4.69)	

ANOVA

According to the results presented in Table 3, there was a significant difference in the mean scores between the 'Total IP' and 'Total Resilience' variables in both the nursing and medicine groups. The participants in both groups reported substantially higher mean scores on the imposter scale (62.4 for nursing students and 61.8 for medicine) compared with their mean scores on the resilience scale (24.3 for nursing and 25.6 for medicine). These findings suggest that individuals in nursing and medicine may experience higher levels of imposter feelings compared to their overall resilience levels. The considerable difference in mean scores (mean difference of 38.05 for nursing and 36.18 for medicine) indicates that imposter feelings are more prevalent in these populations.

**Table 3:** Total mean and SD of imposter and resilience scale among nursing and medical students (*n* = 300)

	*n*	Mean	SD	Mean difference	95% CI		*P*
Total IP	300	62.2	14.63	37.44	35.43	39.45	<0.001
Total Resilience	300	24.8	7.19				
Nursing Total IP Total Resilience	202	62.4	13.64	38.05	35.80	40.30	<0.001
	202	24.3	7.15				
Medicine Total IP Total Resilience	98	61.8	16.55	36.18	32.10	40.27	<0.001
	98	25.6	7.22				

Paired *t*-test

**Figure 4:**
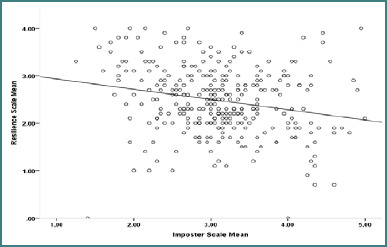
Scatter plot showing the correlation between the Clance Imposter Scale and the Connor-Davidson Resilience Scale

Figure 4 indicates a weak negative correlation between the imposter and resilience scales. This means that the resilience scale scores tend to decrease as the imposter scale scores increase. This negative correlation was statistically significant, with a *P* value less than 0.001, signifying its importance and reliability.

Table 4 displays the correlation between the mean scores of the CIPS scale (3.1 ± 0.73) and the CD-RISC (2.5 ± 0.72). The correlation coefficient between CIPS and CD-RISC was -0.220, indicating a weak negative relationship (*P* < 0.001). This negative correlation suggests that higher CIPS scores were associated with lower CD-RISC scores, implying that resilience tended to decrease as the severity of impostor feelings increased.

**Table 4:** Correlation among Clance Imposter Phenomenon Scale & Connor-Davidson Resilience Scale (*n* = 300)

	*n*	Mean	SD	r	*P*
**Resilience Scale**	300	2.5	0.72	-0.220	<0.001
**Imposter Scale**	300	3.1	0.73		

r=Pearson correlation coefficient

Table 5 presents the association between CIPS and various demographic characteristics. The results indicate that CIPS was significantly influenced by gender, academic specialization, academic year, and socioeconomic status (*P* < 0.05). However, no other significant differences were observed in the remaining variables listed in the table.

**Table 5:** Association of Clance Imposter Phenomenon Scale with demographic characteristics (*n* = 300)

		Clance Imposter Phenomenon Scale								
		Mild		Moderate		Severe		Intense		*P*
		***n* = 23**	**%**	***n* = 113**	**%**	***n* = 125**	**%**	***n* = 39**	**%**	
**Gender**										
	Male	9	(14.5)	20	(32.3)	21	(33.9)	12	(19.4)	0.030*
	Female	14	(5.9)	93	(39.1)	104	(43.7)	27	(11.3)	
**Region**										
	Eastern	5	(13.2)	14	(36.8)	15	(39.5)	4	(10.5)	0.619†
	Western	15	(6.3)	89	(37.6)	101	(42.6)	32	(13.5)	
	Central	2	(13.3)	7	(46.7)	4	(26.7)	2	(13.3)	
	Southern	1	(12.5)	3	(37.5)	4	(50.0)	0	(0.0)	
	Others	0	(0.0)	0	(0.0)	1	(50.0)	1	(50.0)	
**Academic Specification**										
	Nursing	13	(6.4)	77	(38.1)	92	(45.5)	20	(9.9)	0.045*
	Medicine	10	(10.2)	36	(36.7)	33	(33.7)	19	(19.4)	
**Academic Year**										
	3^rd^ year	9	(6.9)	49	(37.4)	53	(40.5)	20	(15.3)	0.041†
	4^th^ year	6	(5.5)	46	(41.8)	49	(44.5)	9	(8.2)	
	5^th^ year	7	(25.0)	8	(28.6)	11	(39.3)	2	(7.1)	
	6^th^ year	1	(3.2)	10	(32.3)	12	(38.7)	8	(25.8)	
**Marital status**										
	Single	21	(7.2)	110	(37.9)	120	(41.4)	39	(13.4)	0.492†
	Married	0	(0.0)	2	(50.0)	2	(50.0)	0	(0.0)	
	Divorced	1	(50.0)	0	(0.0)	1	(50.0)	0	(0.0)	
	Other	1	(25.0)	1	(25.0)	2	(50.0)	0	(0.0)	
**Socioeconomic Status**										
	Low	1	(9.1)	1	(9.1)	6	(54.5)	3	(27.3)	0.001†
	Medium	19	(7.2)	99	(37.4)	117	(44.2)	30	(11.3)	
	High	3	(12.5)	13	(54.2)	2	(8.3)	6	(25.0)	

*Chi-squared test; †Fisher’s exact test; ANOVA

Table 6 displays the relationship between the CD-RISC scale and various demographic characteristics. No statistically significant differences were observed among the variables in the table.

**Table 6:** The association between the Connor-Davidson Resilience Scale and demographic characteristics (*n* = 300)

		Connor-Davidson Resilience Scale								
		1^st^ quartile		2^nd^ quartile		3^rd^ quartile		4^th^ quartile		*P*
		*n* = 221	%	*n* = 35	%	*n* = 26	%	*n* = 18	%	
**Gender**										
	Male	42	(67.7)	7	(11.3)	6	(9.7)	7	(11.3)	0.249*
	Female	179	(75.2)	28	(11.8)	20	(8.4)	11	(4.6)	
**Region**										
	Eastern	27	(71.1)	3	(7.9)	4	(10.5)	4	(10.5)	0.719†
	Western	175	(73.8)	28	(11.8)	22	(9.3)	12	(5.1)	
	Central	12	(80.0)	2	(13.3)	0	(0.0)	1	(6.7)	
	Southern	5	(62.5)	2	(25.0)	0	(0.0)	1	(12.5)	
	Others	2	(100.0)	0	(0.0)	0	(0.0)	0	(0.0)	
**Academic specification**										
	Nursing	151	(74.8)	25	(12.4)	16	(7.9)	10	(5.0)	0.594*
	Medicine	70	(71.4)	10	(10.2)	10	(10.2)	8	(8.2)	
**Academic year**										
	3^rd^ year	94	(71.8)	15	(11.5)	13	(9.9)	9	(6.9)	0.690†
	4^th^ year	86	(78.2)	13	(11.8)	6	(5.5)	5	(4.5)	
	5^th^ year	17	(60.7)	4	(14.3)	4	(14.3)	3	(10.7)	
	6^th^ year	24	(77.4)	3	(9.7)	3	(9.7)	1	(3.2)	
**Marital status**										
	Single	213	(73.4)	34	(11.7)	25	(8.6)	18	(6.2)	0.789†
	Married	3	(75.0)	1	(25.0)	0	(0.0)	0	(0.0)	
	Divorced	2	(100.0)	0	(0.0)	0	(0.0)	0	(0.0)	
	Other	3	(75.0)	0	(0.0)	1	(25.0)	0	(0.0)	
**Socioeconomic status**										
	Low	9	(81.8)	0	(0.0)	2	(18.2)	0	(0.0)	0.050†
	Medium	198	(74.7)	31	(11.7)	23	(8.7)	13	(4.9)	
	High	14	(58.3)	4	(16.7)	1	(4.2)	5	(20.8)	

*Chi-squared test; †Fisher’s exact test; ANOVA

## Discussion

The study aimed to explore the connection between resilience and impostor syndrome in undergraduate nursing and medical students. It aimed to uncover the prevalence and severity of impostor syndrome within the sample. The results showed that the participants had a high mean academic GPA, indicating significant academic achievement. Research by Chong *et al*. [36] indicated that younger medical students had higher levels of imposter phenomenon, while Martin *et al*. [37] discovered a positive correlation between resilience and academic performance among undergraduates. This suggests that younger students may be more vulnerable to impostor syndrome, and there may be a link between resilience and academic success. These findings suggest that younger students may be more vulnerable to impostor syndrome, potentially due to a lack of experience and coping mechanisms that develop with time and maturity, making them more susceptible to feelings of inadequacy and self-doubt. This suggests the need for early interventions focusing on building resilience and coping strategies among first- and second-year students.

Moreover, most participants were women, aligning with previous research that suggests a higher prevalence of the impostor phenomenon among women, potentially due to increased self-doubt and fear of being exposed as a fraud [26]. However, our findings also revealed no significant gender differences in resilience among medical students, suggesting variability across contexts and the need for targeted interventions based on specific demographics. However, it is worth noting that some studies found no significant gender differences in resilience levels among medical students, indicating varied relationships between gender and resilience across contexts [38-40]. The majority of participants with a medium socioeconomic status (88.33%) suggested that socioeconomic status may not significantly impact the imposter phenomenon and resilience levels in this study. This suggests that while socioeconomic status can influence IS, other factors, such as academic environment and support systems, play significant roles. Therefore, policymakers should ensure that resilience-building programs are inclusive and accessible to students from all socioeconomic backgrounds and provide additional support and resources to those from lower socioeconomic status. However, research conducted by Cokley *et al*. [38] among college students found that lower socioeconomic status was associated with higher levels of the imposter phenomenon. These contrasting results indicate that the relationship between socioeconomic status and the imposter phenomenon may vary across different populations and contexts. The impostor syndrome, also known as the impostor phenomenon, can be influenced by cultural factors. For example, women and ethnic minorities often experience impostor feelings due to persistent negative stereotyping, which creates an environment where individuals doubt their abilities and fear being exposed as frauds [39]. While anyone can experience impostor feelings, research consistently suggests that they appear more often in women and people of color [40]. Gender bias and institutionalized racism play a significant part in perpetuating these feelings. Therefore, diverse groups are more vulnerable to impostor syndrome due to discriminatory situations they encounter at schools, workplaces, and communities [41]. These challenges can exacerbate feelings of inadequacy and self-doubt [42]. It is important to note that the current study does not address this diversity, and future research should be directed toward this topic.

The prevalence and severity of impostor syndrome found in this study align with previous research conducted in similar populations. For instance, a study by Park *et al*. [43] among medical students found comparable rates, with 50% of participants reporting high levels of self-doubt and feelings of fraudulence. These findings support the notion that impostor syndrome is a prevalent issue among medical students and can have implications for their well-being and academic performance. In contrast, a study by Smith *et al*. [44] conducted among undergraduate nursing students reported lower rates of impostor syndrome than the current study. Their findings indicated that only 25% of the participants experienced moderate-to-severe IS. This discrepancy in prevalence rates could be attributed to the different measurement tools used or variations in the study samples and cultural contexts. A study by Johnson *et al*. [45] among graduate students from diverse disciplines reported similar proportions of severe (45%) and moderate (36%) IS levels of impostor syndrome. These findings suggest that feelings of inadequacy and self-doubt can be prevalent across different educational contexts, regardless of discipline. On the other hand, a study by Chen *et al*. [46] among undergraduate business students found a higher prevalence of impostor syndrome, with 60% of participants experiencing moderate to severe levels. These contrasting results reflect the unique challenges and expectations of different academic disciplines.

Moreover, the results indicated that less than three-quarters (73.7 %) of the participants fell in the 1^st^ quartile, representing the lowest level of resilience. This finding suggests that a significant proportion of the students in this sample may have lower levels of resilience, potentially impacting their ability to adapt and cope with challenging situations. These results align with those of similar studies conducted among healthcare students. For instance, a study by Jones *et al*. [47] among medical students reported a comparable proportion of students in the lowest quartile of resilience, with 75% in this category. These findings highlight the need for interventions and support programs to enhance resilience among healthcare students.

In contrast, a study by Smith and Lopez [48] among undergraduate nursing students found a higher proportion of students in the highest quartile of resilience compared with the present study. Their results showed that 20% of the participants were in the highest quartile, indicating a relatively high level of resilience among nursing students. Furthermore, a study by Brown *et al*. [49] conducted among graduate students from various fields reported similar resilience distribution patterns. Their findings showed that 10% of the participants fell into the lowest quartile, 20% into the second quartile, 30% into the third quartile, and 40% into the highest quartile.

The study found a weak negative correlation between scores on the CIPS and the CD-RISC scales, indicating that as imposter phenomenon scores increase, resilience scores decrease. This suggests an inverse relationship between imposter phenomenon and resilience among undergraduate nursing and medical students. Similar research supports this negative correlation, while some studies found no significant correlation. These findings imply that individuals experiencing higher levels of imposter phenomenon may struggle with resilience, highlighting the need to address this issue and promote resilience in educational settings [48,49]. There were significant differences in imposter phenomenon scores based on gender and academic specifications. Specifically, the results show a significant difference between women and men (*P* = 0.030). This finding suggests that women may have higher CIPS scale scores than men. This aligns with previous research conducted in various academic and professional contexts. For instance, a study by Auguste, Johnson, and Carr [50] among undergraduate students found that women reported higher levels of the imposter phenomenon compared to men. These results indicate that gender may play a role in the experience of the imposter phenomenon, with women being more susceptible to its effects. Moreover, there was a significant difference in imposter phenomenon scores based on academic specifications (*P* = 0.045). This suggests that students with specific academic specializations may experience different levels of the imposter phenomenon [51]. However, it is challenging to draw specific conclusions without further details or specific academic specifications. The relationship between demographic characteristics and the imposter phenomenon appears complex, with some studies reporting opposite or inconsistent results [52]. Understanding these differences can inform targeted interventions and support systems to address the specific needs of different demographic groups. Further research is recommended to explore the underlying factors contributing to these differences and to develop effective strategies to mitigate the impact of the imposter phenomenon in diverse populations [53,54].

The multinomial logistic regression analysis results indicated that individuals with higher resilience scores were less likely to experience imposter feelings, particularly in the context of mild imposter syndrome. This suggests that higher resilience may help individuals manage feelings of inadequacy and self-doubt, reducing the impact of imposter syndrome. However, as imposter syndrome severity increases to moderate and severe levels, individuals with lower resilience scores are more likely to experience intense imposter feelings. This highlights the critical role of resilience in mitigating the impact of intense imposter feelings. Previous research has also shown that higher levels of resilience are associated with lower levels of the imposter phenomenon [55,56], and resilience has been identified as a protective factor against imposter feelings, enabling individuals to better handle challenges and setbacks in their professional and personal lives [57,58]. It is important to note that the relationship between resilience and the imposter phenomenon can be influenced by factors such as individual personality traits, social support, and cultural context [58-61]. The analysis suggests that higher resilience scores were linked to a reduced likelihood of experiencing imposter feelings, particularly for mild imposter syndrome. Conversely, individuals with lower resilience scores may be more susceptible to experiencing intense imposter feelings as the severity of imposter feelings increases. These findings underscore the importance of promoting resilience as a potential approach to mitigating the impact of imposter syndrome.

The study has limitations, including a small and non-diverse sample, a focus on undergraduate nursing and medical students at one institution, a cross-sectional design of self-reported measures that may have reasons for bias, and a lack of consideration for cultural context. Future research should aim to include a more diversified sample, encompassing multiple universities and varying cultural backgrounds, to provide a broader understanding of imposter syndrome and resilience across different contexts. Longitudinal studies could also explore the long-term effects of resilience training and mentorship programs on impostor syndrome. A deeper investigation is necessary to fully comprehend the intricate interplay between demographic traits and the incidence of impostor syndrome and resilience among undergraduate nursing and medical students. Subsequent studies could delve into the effects of geographical disparities, educational concentration, marital status, academic progression, and economic circumstances on levels of impostor syndrome and resilience. This approach will facilitate a more comprehensive understanding of these variables and support the development of targeted interventions and tailored support networks.

### Research recommendations and implications

Given the findings of the current study, several policy changes and instructional strategies can be recommended to address impostor syndrome and enhance resilience among students:

- Universities should incorporate resilience-building workshops and seminars into the curriculum. These programs can teach coping strategies, stress management, and techniques to boost self-efficacy and confidence among students.
- Establishing mentorship programs where senior students or alumni mentor junior students can provide a support system that helps reduce feelings of impostors. Peer support groups can also be created to foster a sense of community and shared experiences.
- Establishing counseling and psychological services: universities should ensure that counseling services are readily available and promoted among students. Providing access to mental health professionals who can help students manage impostor feelings and build resilience is crucial.
- Creating an inclusive academic environment where diversity is celebrated and all students feel valued can help mitigate impostor feelings. Faculty and staff should be trained to recognize and address impostor syndrome and resilience issues among students.
- Implementing regular assessments of students' mental health and well-being, along with constructive feedback, can help identify those struggling with impostor syndrome early and provide timely interventions.
- Self-confidence and resilience. However, more research is needed to understand how demographic traits affect the incidence of impostor syndrome and resilience among students. This will help institutions create tailored interventions and support networks.

## Conclusion

The results of this study provide valuable insights into the severity levels of impostor syndrome and the levels of resilience among undergraduate nursing and medical students. A significant proportion of students experienced different degrees of impostor syndrome, with many falling into the moderate to severe range. This highlights the importance of implementing interventions and support systems to address impostor syndrome and enhance resilience among these students. While demographic characteristics provide some context, further research is required to understand their effects on impostor syndrome and resilience levels.

## Data Availability

Further data is available from the corresponding author on reasonable request.

## Personal thanks

The researchers would like to acknowledge the support and cooperation of the participants participating in this study. The researchers also expressed gratitude to the educational institution that facilitated the data collection process and provided the necessary study resources.
